# Diagnostic and prognostic value of circular RNAs in hepatocellular carcinoma

**DOI:** 10.1111/jcmm.15258

**Published:** 2020-04-13

**Authors:** Jin‐Yu Sun, Xiao‐Yu Zhang, Yi‐Zhi Cao, Xiao Zhou, Jian Gu, Xiao‐Xin Mu

**Affiliations:** ^1^ Department of General Surgery The First Affiliated Hospital of Nanjing Medical University Sparkfire Scientific Research Group Nanjing Medical University Nanjing China; ^2^ Department of General Surgery Division of Gastrointestinal Surgery Huai'an Second People's Hospital The Affiliated Huai'an Hospital of Xuzhou Medical University Huai'an China; ^3^ Key Laboratory of Liver Transplantation Chinese Academy of Medical Sciences NHC Key Laboratory of Living Donor Liver Transplantation Hepatobiliary Center the First Affiliated Hospital of Nanjing Medical University Nanjing China

**Keywords:** circular RNAs, diagnostic biomarker, hepatocellular carcinoma, prognostic biomarker

## Abstract

Hepatocellular carcinoma (HCC) is the sixth most common malignant tumour, which has posed a heavy health and financial burden worldwide. Due to limited symptoms at the early stage and the limitation in current biomarkers, HCC patients are usually diagnosed at the advanced stage with a pessimistic overall survival rate. Circular RNAs (circRNAs) are a subclass of single‐stranded RNAs characterized by a covalently closed loop structure without 3’‐ or 5’‐end. With advances in high‐throughput sequencing technology and bioinformatics, accumulating studies have demonstrated the promotor or suppressor roles of circRNAs in the carcinogenesis, progression, and metastasis of HCC. Moreover, circRNAs are characteristic of higher abundance, stability and conservation compared with linear RNAs. Therefore, circRNAs have emerged as one of the most promising diagnostic and prognostic biomarkers for HCC with reliable accuracy, sensitivity and specificity. In this review, we briefly introduce the characteristics of circRNAs and summarize the roles of circRNAs in the biological procedures of HCC. Furthermore, we provide an overview on the potential diagnostic and prognostic value of circRNAs as biomarkers for patients with HCC. Finally, we discuss future perspectives of circRNAs in cancer research.

## 1 INTRODUCTION

Hepatocellular carcinoma (HCC) is the sixth most common malignant tumour with an increasing incidence.^1^, ^2^ As a major subtype of primary liver cancer, HCC accounts for approximately 90% in general and is now the fourth leading cause of cancer‐related death worldwide, imposing severe health and financial burden.^2^, ^3^ Current treatment strategies for HCC include hepatic resection, percutaneous thermal ablation, radiotherapy, systemic treatment and immunotherapy.^4^ However, since HCC patients show limited symptoms at the early stage, more than 75% of HCC patients are diagnosed at the advanced stage with tumour cell metastasis or diffusion.^5^ Also, the treatment response of patients in the advanced stage is usually poor, and they commonly suffer from a low survival rate. Hence, it is essential to diagnose HCC at the early stage, and there is an urgent need to develop novel diagnostic or prognostic biomarkers for HCC.

Circular RNAs (circRNAs) are a subclass of single‐stranded RNAs characterized by a covalently closed loop structure. Base on whether they can be translated, circRNAs can be divided into non‐coding circRNAs and coding circRNAs.^6^ With the advances in high‐throughput sequencing technology and bioinformatics, a variety of circRNAs have been demonstrated to play essential roles in regulating gene expression at transcriptional or post‐transcriptional levels^7^, ^8^, ^9^, ^10^ and participate in multiple biological processes of HCC.^11^ Moreover, owing to the single‐stranded closed circular structure, circRNAs show higher abundance, stability and conservation compared with linear RNAs. These advantages make circRNAs one of the most promising biomarkers in the diagnosis and prognosis of HCC.^12^, ^13^

In this review, we briefly introduce the characteristics of circRNAs and summarize their roles in the biological processes of HCC. Furthermore, we provide an overview on the potential diagnostic and prognostic value of circRNAs as biomarkers. Finally, we give an insight into future perspectives of circRNAs in cancer research.

## 2 CHARACTERISTICS OF CIRCRNAS

CircRNAs were initially discovered as aberrant by‐products or abnormally spliced transcripts in human cells, while their specific roles remained vague due to the limitation of traditional RNA sequencing technologies.^14^, ^15^ Owing to the improvement of specialized computational pipelines in the last decade, accumulating information has been gathered in this area, and the roles of circRNAs in various diseases are becoming increasingly evident.^16^, ^17^, ^18^, ^19^

**Figure 1:**
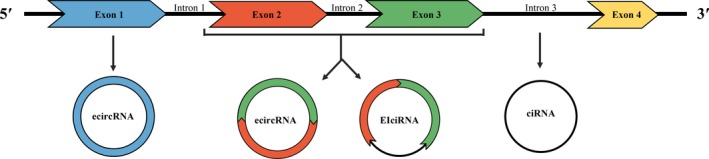
The biogenesis of circRNAs. CircRNAs are single‐stranded transcripts arising from exons (ecircRNAs), introns (ciRNAs), or both exons and introns (EIciRNAs)

CircRNAs are single‐stranded transcripts arisen from exons (ecircRNAs),^20^ introns (ciRNAs)^21^ or both (EIciRNAs)^18^, ^22^ (Figure 1). CircRNAs are extensively expressed in eukaryotic cells and characterized by high abundance, stability, conservation, as well as a tissue‐ or developmental‐specific expression pattern.^11^, ^23^, ^24^, ^25^ The covalently closed circular structure makes circRNAs more stable and resistant to exonuclease‐induced degradation compared with linear RNAs.^26^ Moreover, circRNAs display evolutionary conservation across multiple species and the expression profiles are tissue‐ or developmental stage‐specific, suggesting the broad participation in various physiological and pathophysiological processes.^23^, ^27^, ^28^, ^29^

Accumulating evidence has revealed the function of circRNAs in regulating gene transcription and expression, and the miRNA sponge activity is the hallmark function of circRNAs.^30^ CircRNAs have a significant number of binding sites for miRNAs and can act as sponges of miRNAs, which would subsequently influence the stability and translation of target RNAs.^31^ Apart from miRNA sponge activity, circRNAs can also interact with RNA binding proteins,^32^ function as transcriptional or translational regulators,^22^ influence splicing of pre‐mRNAs ^33^ and participate in protein translation.^34^, ^35^

## 3 ROLES OF CIRCRNAS IN HCC

Recently, the knowledge of circRNAs is accumulating at an increasing pace, and plenty of studies have revealed the association between circRNAs and various biological processes in cancers, including cell proliferation, apoptosis, invasion, as well as metastasis. In HCC, numerous circRNAs are dysregulated in tumour tissues and may play oncogenic or suppressor roles in cancer development and progression.^36^, ^37^, ^38^, ^39^, ^40^, ^41^, ^42^, ^43^ Generally, most of the up‐regulated circRNAs are positively associated with HCC progression, whereas down‐regulated circRNAs usually act as suppressors preventing the development of HCC. Table 1 summarizes recent studies on the regulating effect of circRNAs in HCC.^44^, ^45^, ^46^, ^47^, ^48^, ^49^, ^50^, ^51^, ^52^, ^53^, ^54^

**Table 1:** Recent studies on the regulating effect of circRNAs in HCC

circBase ID (Alias)	Alteration	Target	Function	Ref.
hsa_circ_0001946 (Cdr1as/ciRS‐7)	↑	miR‐7	Oncogene	55, 56, 58
hsa_circRNA_101368	↑	HMGB1/RAGE	Modulate the migration of HCC	^42^
Circ‐CDYL	↑	miR‐892a and miR‐328‐3p	Oncogene	^9^
hsa_circRNA‐104718	↑	miR‐218‐5p/TXNDC5	Promote HCC progression	^54^
hsa_circ_0067934	↑	miR‐1324/FZD5/Wnt/β‐catenin	Promote tumour growth and metastasis	^60^
hsa_circ_0015756	↑	miR‐7	Promote proliferation, invasion and migration	^61^
hsa_circ_0020007 (circ‐ADD3)	↓	EZH2	Inhibit HCC metastasis	^63^
hsa_circ_0001445 (cSMARCA5)	↓	miR‐17‐3p and miR‐181b‐5p	Inhibit growth and metastasis in HCC	^10^
hsa_circ_0000284 (circHIPK3)	↑	miR‐124	Regulate cell proliferation and migration	^44^
hsa_circ_0000567 (circSETD3)	↓	miR‐421	Inhibit the proliferation of HCC cells	^8^
hsa_circ_0000847 (circSMAD2)	↓	miR‐629	Inhibit the migration and epithelial‐mesenchymal transition of HCC cells.	^45^
hsa_circ_0016788	↑	miR‐486/CDK4	Oncogene	^59^
hsa_circ_0128298	↑	‐	Promote proliferation and metastasis	^72^
hsa_circ_0001727 (circZKSCAN1)	↓	Multiple cancer‐related signalling pathways	Inhibit HCC cell growth, migration and invasion	^48^
hsa_circ_0007144 (circPTPRM)	↑	‐	Promote proliferation and migration	^46^
hsa_circRNA8662‐12 (circTRIM33‐12)	↓	miR‐191	Inhibit HCC proliferation, metastasis and immune evasion	^64^
hsa_circ_0002768 (circRNA‐MYLK)	↑	miR‐362‐3p	Promote the proliferation, invasion and migration	^47^
hsa_circRNA_102034 (circRHOT1)	↑	NR2F6	Promote HCC growth and metastasis	^49^
hsa_circRNA_103809	↑	miR‐377‐3p	Promote the proliferation, cycle progression and migration of HCC cells	^36^
hsa_circ_0001649	↓	miR‐127‐5p, miR‐612 and miR‐4688	Inhibit proliferation and migration of HCC	^50^
hsa_circ_001013 (circHIAT1)	↓	miR‐3171	Inhibit the growth of HCC cells	^51^
Circβ‐catenin	↑	Wnt pathway	Promote HCC development	^52^
hsa_circ_0000267	↑	miR‐646	Facilitate cell growth, migration and invasion	^75^
hsa_circ_0008450	↑	miR‐548p	Promote cell viability, migration and invasion	^37^
CircADAMTS13	↓	miR‐484	Suppress cell proliferation	^38^
hsa_circRNA_104075	↑	miR‐582‐3p	Promote HCC development	^39^
hsa_circ_0018665 (circADAMTS14)	↓	miR‐572	Inhibit tumour growth	^40^
hsa_circ_101280	↑	miR‐375	Promote HCC	^41^
hsa_circ_0016788	↑	miR‐486	Promote the proliferation, invasion and inhibit the apoptosis	^59^
Circ‐FOXP1	↑	miR‐875‐3p and miR‐421	Promote HCC progression	^53^
hsa‐circ‐0046600	↑	miR‐640/HIF‐1α	Promote HCC progression	^43^

Abbreviations: ‐, not provided; ↑, Up‐regulated; ↓, Down‐regulated; HCC, hepatocellular carcinoma.

Cdr1as (hsa_circ_0001946), a sponge of miR‐7, is one of the classic circRNAs acting as a promotor in the progression of HCC. Cdr1as was reported to be significantly up‐regulated in HCC cell lines and HCC tissues compared with the non‐tumour ones, and the expression level of Cdr1as was positively associated with hepatic microvascular invasion as well as deterioration.^55^, ^56^ As a target of Cdr1as, miR‐7 targets several oncogenes directly, and the up‐regulation of miR‐7 inhibits the proliferation and invasion of HCC cells.^57^ Moreover, knockdown of Cdr1as would suppress the HCC cell proliferation and invasion,^56^ while overexpression of Cdr1as could lead to the opposite effect.^58^ Importantly, exosomes acquired from HCC cells overexpressing Cdr1as could also accelerate the proliferation and migration of surrounding normal cells.^58^

Guan et al^59^ conducted circRNA microarray analysis in three pairs of HCC and adjacent healthy tissues. Hsa_circ_0016788 was significantly up‐regulated both in HCC tissue and cell lines. The silence of hsa_circ_0016788 could retard tumour growth and inhibit the proliferation and invasion of tumour cells. Also, the bioinformatics analysis showed that hsa_circ_0016788 accelerated HCC progression via miR‐486/CDK4. These results indicated the oncogenic role of hsa_circ_0016788 in HCC.

Moreover, Wei et al^9^ revealed a circRNA‐centric non‐coding regulatory RNA network activated in the early stage of HCC based on RNA expression profiles. Circ‐CDYL was reported to be highly expressed in the early stage of HCC, which promoted the properties of epithelial cell adhesion molecule‐positive liver tumour‐initiating cells. Additionally, circ‐CDYL could promote the expression of several proto‐oncogenes via PI3K‐AKT‐mTORC1/β‐catenin and NOTCH2 pathways in HCC cells. Similarly, various circRNAs were also reported as promotors for carcinogenesis and cancer progression, such as hsa_circ_0067934,^60^ circHIPK3 (hsa_circ_0000284),^44^ hsa_circ_0015756,^61^ hsa_circ_0001955 ^62^ and so forth.

Apart from oncogenic roles, circRNAs could also act as tumour suppressors preventing the occurrence and development of HCC. For example, Sun et al^63^ showed a significant decrease of circ‐ADD3 (hsa_circ_0020007) in HCC by circRNA microarray expression profile in matched HCC and para‐cancerous tissues. Increased expression of circ‐ADD3 could effectively weaken the vascular invasion, intrahepatic metastasis and distant metastasis of HCC via regulating EZH2 stability. Moreover, Zhang et al^64^ demonstrated that circTRIM33‐12(has_circRNA8662‐12), a sponge of miR‐191, was down‐regulated in HCC tissues and cell lines, and the reduced expression of circTRIM33‐12 could significantly promote tumour proliferation, migration, invasion as well as immune evasion abilities of HCC cells.

## 4 DIAGNOSTIC VALUE OF CIRCRNAS IN HCC

Due to limited symptoms at the early stage and the limitation in current biomarkers, HCC patients are usually diagnosed at the advanced stage with a pessimistic overall survival rate.^65^ Moreover, current biomarkers, such as α‐fetoprotein (AFP) and AFP‐L3, show modest diagnostic performance for HCC.^65^ Since circRNAs are closely associated with multiple biological processes in HCC and characteristic with high stability and abundance in HCC tissue as well as in body fluid, circRNAs have been proposed as diagnostic biomarkers for HCC. For example, Yao et al^66^ revealed the close association between hsa_circ_0068669 expression level and microvascular invasion, and they demonstrated hsa_circ_0068669 as a potential biomarker for HCC metastasis. Table 2 summarizes recent studies on circRNAs as diagnostic biomarkers for HCC.^67^, ^68^, ^69^

**Table 2:** Recent studies on circRNAs as diagnostic biomarkers of HCC

circBase ID (Alias)	Gene symbol	Genomic position	AUC	Sensitivity (%)	Specificity (%)	Ref.
Circ‐CDYL, plus HDGF and HIF1AN	‐	‐	0.73	75.4	66.67	^9^
hsa_circ_001565	B4GALT2	chr1:44446997‐44447136	‐	73.5	82.29	^71^
hsa_circ_000224	C17orf107	chr17:4803230‐4803902	‐	95.6	92.7	^71^
hsa_circ_000520	VIM	chr10:17271723‐17271867	‐	97.1	89.6	^71^
hsa_circ_0016788	TRIM11	chr1:228581376‐228594517	0.851	‐	‐	^59^
hsa_circ_0128298	SPINK1	chr5:147210311‐147211162	0.668	71.6	81.5	^72^
hsa_circ_0001727 (CirZKSCAN1)	ZKSCAN1	chr7:99621041‐99621930	0.834	82.2	72.4	^48^
hsa_circ_0000976, plus hsa_circ_0007750 and hsa_circ_0139897	HPCAL1, RABGGTA, and MTM1, respectively	chr2:10559859‐10560261, chr14:24735635‐24737825, and chrX:149761066‐149787612, respectively	0.843	87.5	81.2	^73^
hsa_circRNA_104075	NUP153	chr6:17669524‐17669777	0.973	96.0	98.3	^39^
hsa_circ_0005075	EIF4G3	chr1:21377358‐21415706	0.94	83.3	90.0	^67^
hsa_circ_0028502	SLC24A6	chr12:113753139‐113754806	0.675	72.1	58.0	^70^
hsa_circ_0076251	ZFAND3	chr6:38050167‐38084515	0.738	71.3	64.0	^70^
hsa_circ_0027089	PTGES3	chr12:57059987‐57064148	0.784	57.8	84.8	^68^
hsa_circ_0003998	ARFGEF2	chr20:47570092‐47580435	0.894	84.0	80.0	^69^

Abbreviations: ‐, not provided; AUC, area under the curve.

Circ‐CDYL is a promotor for HCC, and its up‐regulation could increase the expression of several proto‐oncogenes. Recently, Wei et al^9^ showed the diagnostic performance of circ‐CDYL in the early stage of HCC with an area under the curve (AUC) of 0.64 (95% CI = 0.55‐0.72). When comprehensively analysing the expression levels of circ‐CDYL plus HDGF and HIF1A, the results showed an improved diagnostic performance with an AUC of 0.73 (95% CI = 0.65‐0.80), a sensitivity of 75.36% and specificity of 66.67%. Compared with circ‐CDYL plus HDGF and HIF1A, AFP only showed an AUC of 0.59 (95% CI = 0.49‐0.70), a sensitivity of 50.72% and a specificity of 83.78%. This study indicated that circ‐CDYL plus HDGF and HIF1AN might be more reliable diagnostic biomarkers than AFP.

Jiang et al^70^ analysed the expression levels of hsa_circ_0028502 and hsa_circ_0076251 in cancer and adjacent para‐cancerous tissues. The results showed that both the hsa_circ_0028502 and hsa_circ_0076251 levels were significantly lower in HCC tissues (*P* < .001). Importantly, hsa_circ_0028502 level was related to tumour node metastasis stage (*P* = .015), while hsa_circ_0076251 expression was related to Barcelona Clinic Liver Cancer stage (*P* = .038). When distinguishing HCC tissues from the liver cirrhosis tissues and chronic hepatitis tissues, the AUCs of hsa_circ_0028502 and hsa_circ_0076251 were 0.675 and 0.738, respectively. Additionally, Matboli et al^71^ assessed the diagnostic performance of hsa_circ_001565, hsa_circ_000224 and hsa_circ_000520 for HCC, and the results showed a higher sensitivity and specificity compared with AFP. When combined with these three biomarkers, the diagnostic performance was further improved to an accuracy of 80.49%, a sensitivity of 100%, a specificity of 83.3%. Moreover, the receiver operating characteristic (ROC) curve analysis of hsa_circ_0016788 was performed based on 40 HCC patients and 40 healthy controls, and the AUC of 0.851 was acquired.^59^ Similarly, hsa_circ_0128298, which was significantly up‐regulated in HCC tissues, showed an AUC of 0.668, a sensitivity of 0.716 and specificity of 0.815.^72^

Furthermore, Yao et al^48^ analysed the expression of circZKSCAN1 (hsa_circ_0001727) in a cohort of 102 patients with HCC, and its expression was significantly lower in tumour tissues than matched adjacent non‐tumorous samples (*P* < .05). Importantly, circZKSCAN1 level was associated with various clinical characteristics, including tumour numbers (*P* < .01), cirrhosis (*P* = .031), vascular invasion (*P* = .002), microvascular invasion (*P* = .002) and tumour grade (*P* < .001). Also, as a diagnostic biomarker, circZKSCAN1 showed reliable performance with an AUC of 0.834, a sensitivity of 82.2% and specificity of 72.4%.

Apart from HCC tissue, the alteration of circRNAs in plasma could also be used as diagnostic biomarkers for HCC. In a large‐scale multicenter study, Yu et al^73^ designed and evaluated a plasma circRNA panel (circPanel) consisting of hsa_circ_0000976, hsa_circ_0007750 and hsa_circ_0139897 to diagnose HBV‐related HCC. The newly created circPanel showed a better diagnostic performance (AUC = 0.843, 95% CI = 0.796‐0.890) than AFP (AUC = 0.747, 95% CI = 0.691‐0.804) in the validation set containing 306 individuals. Moreover, circPanel also showed a reliable performance in diagnosing small‐HCC (solitary ≤ 3 cm) and AFP‐negative HCC with AUCs of 0.838 (95% CI = 0.776‐0.900) and 0.857 (95% CI = 0.793‐0.921), respectively.

## 5 PROGNOSTIC VALUE OF CIRCRNAS IN HCC

Since circRNAs are involved in multiple biological processes in HCC, the prognostic value of circRNAs also attracts wide attention. Table 3 summarizes recent studies on circRNAs as prognostic biomarkers of HCC.

**Table 3:** Recent studies on circRNAs as prognostic biomarkers of HCC

circBase ID (Alias)	Gene symbol	Genomic position	Prognosis	Univariate analysis			Multivariate analysis			Ref.
				HR	95% CI	*P*	HR	95% CI	*P*	
hsa_circ_0001445 (cSMARCA5)	SMARCA5	chr4:144464661‐144465125	OS	‐	‐	<.001	2.47	1.46‐4.18	.001	^10^
			RFS	‐	‐	.001	1.67	1.08‐2.59	.021	^10^
hsa_circ_0001946 (Cdr1as/ciRS‐7)	CDR1	chrX:139865339‐139866824	MVI	2.65	1.06‐6.63	.037	4.08	1.06‐15.74	.041	^55^
hsa_circ_0128298	SPINK1	chr5:147210311‐147211162	OS	1.98	1.34‐3.02	.009	6.66	2.66‐8.42	.014	^72^
hsa_circRNA8662‐12 (circTRIM33‐12)	‐	‐	OS	‐	‐	.001	0.50	0.93‐1.94	.007	^64^
hsa_circ_0001727 (circZKSCAN1)	ZKSCAN1	chr7:99621041‐99621930	OS	0.09	10.04‐0.19	<.001	0.10	0.05‐0.23	<.001	^32^
			RFS	0.22	0.12‐0.41	<.001	0.22	0.11‐0.42	<.001	^32^
hsa_circ_0000267	FAM53B	chr10:126370175‐126370948	OS	2.64	1.43‐4.87	.002	2.11	1.09‐4.06	.025	^75^
Hsa_circ_0076251	ZFAND3	chr6:38050167‐38084515	OS	0.46	0.22‐0.98	<.05	‐	‐	‐	^70^
Hsa_circ_0003998	ARFGEF2	chr20:47570092‐47580435	OS	0.60	0.42‐0.86	.006	0.58	0.41‐0.84	.003	^69^

Abbreviations: ‐, not provided; CI, concordance index; HR, hazard ratios; MVI, microvascular invasion; OS, overall survival; RFS, relapse‐free survival.

A study on 112 patients with HCC showed that circZKSCAN1 expression was closely associated with various HCC characteristics. In the Kaplan‐Meier survival analysis, the expression level of circZKSCAN1 in cancer tissues was positively correlated with HCC prognosis (overall survival, *P* < .001). The further univariate and multivariate analysis showed that circZKSCAN1 expression was an independent factor for overall survival rate (HR = 0.104; 95% CI: 0.046‐0.234, *P* < .001) as well as RFS rate (HR = 0.219; 95% CI: 0.114‐0.420, *P* < .001) for HCC patients.^32^

Moreover, Matboli et al^71^ demonstrated that HCC patients with negative hsa_circ_001565, hsa_circ_000224 or hsa_circ_000520 had relatively more prolonged relapse‐free survival (RFS) after a median follow‐up of 26 months. Moreover, Kaplan‐Meier analysis suggested a significant decrease in RFS and an increase in cumulative hazards among hsa_circ_000520 in patients with HCC. And the Cox multivariate analysis indicated the expression level of hsa_circ_000520 as an independent prognostic factor of RFS.^71^ Similarly, low expression of circTRIM33‐12 was closely correlated with poor prognosis and was demonstrated as an independent predictor for OS (*P* = .001 in univariate analysis; *P* = .007 in multivariate analysis) as well as post‐operative recurrence (*P* = .001 in univariate analysis; *P* = .005 in multivariate analysis).^64^

In addition, circASAP1, a competing endogenous RNA for miR‐326 and miR‐532‐5p, was closely associated with pulmonary metastasis of HCC after curative resection.^74^ In vitro, the overexpressed circASAP1 could promote cell proliferation, migration and invasion, as well as enhancing tumour growth and pulmonary metastasis in vivo. The analysis of clinical tumour samples showed a positive association between up‐regulated circASAP1 and tumour‐associated macrophages (eg CSF‐1 and MAPK1).

Besides, Yu et al^10^ demonstrated that cSMARCA5 (hsa_circ_0001445) could inhibit the proliferation and migration of HCC cells, and down‐regulated cSMARCA5 expression was significantly correlated with aggressive clinicopathological characteristics. The Kaplan‐Meier survival analysis suggested that HCC patients with low cSMARCA5 level had a significantly reduced overall survival (*P* = .0004) and RFS (*P* = .0008). Further univariate and multivariate analysis also indicated that the cSMARCA5 level was an independent risk factor for overall survival and RFS after hepatectomy.^10^ Similarly, the prognostic ability of hsa_circ_0128298 was evaluated by Cox regression analysis, and the results showed that the expression level of hsa_circ_0128298 could be used as a prognostic factor to predict poor OS in HCC patients (*P* = .009 in univariate analysis; *P* = .014 in multivariate analysis).^72^ The Kaplan‐Meier survival analysis also showed a statistically better overall survival in patients with low expression of hsa_circ_0128298 compared to those with high expression (*P* = .003).^72^

CircRHOT1 (hsa_circRNA_102034), which was prominently up‐regulated in HCC tissues, was demonstrated to promote tumour growth and metastasis significantly,^49^ and patients with high‐level expression of circRHOT1 were associated with a more reduced overall survival rate (*P* = .02) and recurrence‐free survival rate (*P* = .02).^49^ Moreover, hsa_circ_0000267 was up‐regulated in HCC tissue and cell line, and it was considered as a critical oncogene to facilitate the initiation and progression of HCC. In the multivariable analysis performed by Pan et al^75^ the results suggested hsa_circ_0000267 could be used as an independent prognostic indicator for overall survival (HR = 2.107; 95% CI: 1.093‐4.064,* P* = .025).

## 6 CONCLUSIONS AND FUTURE PERSPECTIVES

HCC is the sixth most common malignant tumour with a pessimistic survival,^76^ which accounts for approximately 90% of primary liver cancers. Due to limited symptoms at the early stage and the lack of satisfying biomarkers, more than 75% of HCC patients are diagnosed at the advanced stage with a reduced overall survival rate.^65^ Despite the advances in treatment strategies against HCC, such limitation makes HCC remain one of the most lethal cancers, which accounts for at least 700,000 deaths worldwide annually.^77^, ^78^ Numerous circRNAs have been found to be dysregulated in tumour tissues, and accumulating studies have demonstrated the oncogenic or suppressor roles of circRNAs in the carcinogenesis and progression of HCC.^79^ Moreover, circRNAs are characteristic of higher abundance, stability and conservation compared with linear RNAs. Therefore, circRNAs have recently emerged as one of the most promising diagnostic and prognostic biomarkers for HCC.

However, the research of circRNAs in HCC remains in its infancy stage, the mechanisms underlying the contribution of circRNAs to HCC generation and progression remain vague, and their overall function has not yet been fully understood. Compared with miRNAs or lncRNAs,^80^ only a small amount of functional circRNAs have been discovered in HCC. So far, multiple theories have been proposed, and circRNAs may serve their functions in carcinogenesis, progression and metastasis by various mechanisms. The improved understanding of the molecular mechanisms and associated signalling pathways of these functional circRNAs will positively facilitate the identification of biomarkers and even therapeutic targets for HCC. In the next step, it is essential to study the function of circRNAs in HCC thoroughly and uncover the exact mechanisms of how circRNAs promote or suppress the progression of HCC, which will accelerate the clinical application of circRNAs in the diagnosis, prognosis and treatment of HCC.

Moreover, the number of HCC patients recruited in the diagnosing test is relatively small, and therefore, the results may be over‐interpreted. More studies on a large cohort are needed to validate the actual diagnostic or prognostic effect of circRNAs.

This review primarily provides an overview of circRNAs with a focus on the diagnostic or prognostic value, while the application of circRNAs as therapeutic targets has also attracted wide attention. For example, the knockdown of Cdr1as ^56^ or circ‐TCF4.85 ^81^ was demonstrated to suppress the proliferation and invasion of HCC cells. Further, the subcutaneous injection of si‐circ‐TCF4.85‐transfected HCC cells could inhibit xenograft tumour formation in nude mice, which suggested the potential roles of circRNAs in cancer treatment. With the accumulating findings, circRNAs might be further applied in HCC as therapeutic targets in the future.

## CONFLICT OF INTEREST

The authors confirm that there are no conflicts of interest.

## AUTHOR CONTRIBUTION

Jin‐Yu Sun, Xiao‐Yu Zhang, Xiao Zhou and Xiao‐Xin Mu involved in conceptualization. Jin‐Yu Sun and Yi‐Zhi Cao involved in writing‐original draft. Jian Gu and Xiao‐Xin Mu involved in writing‐review and editing.
